# Robotic Training Using a Novel Upper-Limb Hybrid Assistive Limb After Brachial Plexus Injury: An Electrophysiological Observational Case Series Study

**DOI:** 10.3390/jfmk11030332

**Published:** 2026-08-25

**Authors:** Shigeki Kubota, Hideki Kadone, Yukiyo Shimizu, Masashi Yamazaki

**Affiliations:** 1 Department of Orthopaedic Surgery, Institute of Medicine, University of Tsukuba, 1-1-1 Tennodai, Tsukuba 305-8575, Ibaraki, Japan; masashiy@tsukuba-seikei.jp; 2 Department of Occupational Therapy, Ibaraki Prefectural University of Health Sciences, 4669-2 Ami, Ami, Inashiki 300-0394, Ibaraki, Japan; 3 Center for Innovating Medicine and Engineering (CIME), University of Tsukuba, 1-1-1 Tennodai, Tsukuba 305-8575, Ibaraki, Japan; kadone@md.tsukuba.ac.jp; 4 Department of Rehabilitation Medicine, Institute of Medicine, University of Tsukuba, 1-1-1 Tennodai, Tsukuba 305-8575, Ibaraki, Japan; shimiyukig@md.tsukuba.ac.jp

**Keywords:** traumatic brachial plexus injury, intercostal nerve transfer, musculocutaneous nerve, electrophysiology

## Abstract

**Background and Objectives:** The hybrid assistive limb (HAL) is a wearable robotic device used for rehabilitation that assists the voluntary movements of the user by detecting muscle action potentials and driving actuators positioned next to the hip and knee joints. Although upper-limb HAL training has been studied for brachial plexus injury (BPI), its electrophysiological influence remains unclear. The purpose of this study was to assess the electrophysiological influences of upper-limb HAL-assisted biofeedback (BF) training during elbow flexion rehabilitation in patients with BPI. **Methods:** Five patients with BPI (average age, 37.2 years) were enrolled after undergoing elbow flexor reconstruction through intercostal nerve-to-musculocutaneous nerve transfer. All participants received outpatient elbow flexion training with the upper-limb HAL at frequencies ranging from once weekly to once monthly. All patients started upper-limb HAL training when re-innervation was observed, and a biceps brachii muscle strength of grade 1 was achieved. Muscle activity was measured using surface electromyography in five patients during upper-limb HAL training, when the biceps brachii muscle strength was graded as Medical Research Council grades 1 and 2, to compare activity with and without HAL. **Results:** In five patients, electromyographic activity of the biceps brachii during elbow flexion reached 74.9 ± 22.7% of maximal contraction while using the HAL device, compared with 60.3 ± 16.7% without HAL assistance, indicating significantly greater muscle activation during HAL-assisted movement. **Conclusions:** Robotic BF training for elbow flexion with the upper-limb HAL may serve as a high-quality electromyographic rehabilitation approach for patients recovering from BPI.

## 1. Introduction

Traumatic brachial plexus injury (BPI) is a severe peripheral nerve palsy that results in upper-limb dysfunction [1]. Serious BPI in adults predominantly involves high-energy injuries caused by motorcycle accidents [2]. Surgical options for restoring elbow flexion after BPI include neurolysis, nerve transfer, nerve grafting, and free muscle transfer [3,4]. Intercostal nerve-to-musculocutaneous nerve (ICN-MCN) transfer is commonly performed for restoration of elbow flexion in patients with BPI. Postoperative antigravity elbow flexion can be achieved in approximately 60–90% of cases [5,6,7,8,9,10]. However, satisfactory voluntary elbow flexion is not obtained in all patients after surgery [11]. Various factors have been associated with suboptimal recovery, including patient age, the interval from injury to surgical intervention, and operative technique; in addition, the quality and duration of postoperative rehabilitation are important contributors to functional outcomes [11,12,13]. It usually takes several months to a year for a patient to achieve re-innervation and contraction of the biceps brachii after intercostal nerve transfer and 1–2 years until the patient can flex the elbow against gravity. This is often followed by 2–3 years of long-term rehabilitation.

Conventional biofeedback (BF) therapy is initiated when biceps brachii contraction is observed after intercostal nerve transfer (post-reinnervation) through motor control of the biceps brachii re-innervated by the intercostal nerve. The goal of conventional BF therapy is to re-establish voluntary elbow flexion independent of breathing, which is mediated by the intercostal nerve [14]. However, treatment effects are evaluated using only surface electrodes attached to the re-innervated muscle to display the contraction waveform on a monitor. This is a limitation of conventional therapy, as actual elbow movements are not observed. Improvements in this conventional method and the establishment of novel BF therapy would help develop more useful elbow flexor power.

Recent advances in upper-limb robotic technologies for rehabilitation have enabled the implementation of intensive, repetitive training programs on a broader scale [15,16,17,18,19,20]. The hybrid assistive limb (HAL) is a wearable rehabilitation robot that identifies muscle-generated bioelectric signals from the skin surface of the user and supports voluntary motion through actuators positioned on the lateral aspects of the hip and knee joints [21]. We have been performing robotic rehabilitation of the elbow and shoulder using a novel single-joint HAL in patients with upper-limb disabilities, including those associated with BPI [22,23,24]. The upper-limb HAL uses bioelectric signals detected by surface electrodes placed over the biceps and triceps brachii muscles to provide real-time support for voluntary elbow flexion and extension. This assistance can be achieved even in patients with severely decreased muscle strength (Medical Research Council [MRC] grade 1 or 2) [25], where the bioelectric activity alone is insufficient to generate joint movement.

With regard to rehabilitation after intercostal nerve transfer for BPI, conventional audio-visual electromyography (EMG) BF therapy can also start with an MRC grade 1 with reinnervation of the biceps after intercostal nerve transfer. Patients receiving this therapy can see and hear the EMG waveform on a monitor and the sounds from their biceps that were recorded through a surface electrode. However, the conventional audio-visual EMG BF therapy cannot create voluntary elbow motion. Because BF therapy can enable elbow flexion in patients with a strength of MRC grade 1, it may also be able to support visual input from real elbow flexion, thereby affecting the proprioceptors in the muscle spindle (deep sensibility), and could enable repetitive joint motion training with real muscle contraction earlier after surgery. Hence, training with the upper-limb HAL may accelerate the achievement of voluntary motion of a strength of MRC grade 3 by stimulating changes in the central nervous system following intercostal nerve transfer.

Thus, elbow muscle training with the novel upper-limb HAL can help in the early recovery and reacquisition of stronger elbow flexor power in patients post-BPI and post-elbow flexor reconstruction. Using the upper-limb HAL allows training to start from the early postoperative period, when elbow flexion is not yet possible (biceps MRC grade 1 or 2), and such a training program may aid in good recovery. Although upper-limb HAL training has been studied for BPI [11,22,24], its electrophysiological influences remain unclear and have not yet been reported. Therefore, in this study, we aimed to evaluate the electrophysiological influences of upper-limb HAL-assisted elbow flexion BF training in patients with BPI.

## 2. Materials and Methods

### 2.1. Study Design

In this single-arm study, we investigated the electrophysiological influences of upper-limb HAL training in individuals with BPI. Elbow-joint training using the HAL system (CYBERDYNE, Inc., Tsukuba, Ibaraki, Japan) was conducted during the postoperative rehabilitation period following BPI surgery. The study protocol received approval from the Ethics Committee of our university (TCRB18-38). All participants were fully informed of the objectives and methodology of the study and provided written informed consent for both participation and publication. The study was conducted according to the principles of the World Medical Association (WMA) Declaration of Helsinki—Ethical Principles for Medical Research Involving Human Subjects, following the amendments made in Seoul, South Korea, in October 2008, with a note of clarification on paragraph 29 added by the WMA General Assembly in Washington (2002) and a note of clarification on paragraph 30 added by the WMA General Assembly in Tokyo (2004). This study was also conducted in accordance with the Japanese Medical Research Involving Human Subjects Act and other guidelines, regulations, and acts.

### 2.2. Inclusion and Exclusion Criteria

Patients were included if they (1) had a previous diagnosis of BPI and underwent surgery or conservative therapy; (2) understood the study explanation and provided informed consent; (3) had a body size that could fit in the upper-limb HAL; and (4) were able to participate in standard postoperative physical and occupational therapy programs. Patients were excluded if they had (1) inadequately controlled cardiovascular disorders, (2) inadequately controlled respiratory disorders, (3) intellectual impairments limiting the comprehension of instructions, (4) moderate-to-severe joint disorders, including elbow contractures, and (5) moderate-to-severe involuntary movements (e.g., ataxia) or impaired postural reflexes of the trunk or upper limbs.

### 2.3. Patients

Five patients with BPI, including five men, were enrolled in this study. All injuries resulted from traffic accidents. Regarding the injury pattern, four patients had whole-type BPI, one had a C5–8 type injury. Every patient underwent elbow flexor reconstruction using ICN-MCN transfer. Upper-limb HAL training for elbow flexion was initiated an average of 8.6 months after surgery (range, 8–9 months), and rehabilitation sessions were performed on an outpatient basis at frequencies ranging from once weekly to once monthly.

### 2.4. Upper-Limb HAL Training Program

**Figure 1:**
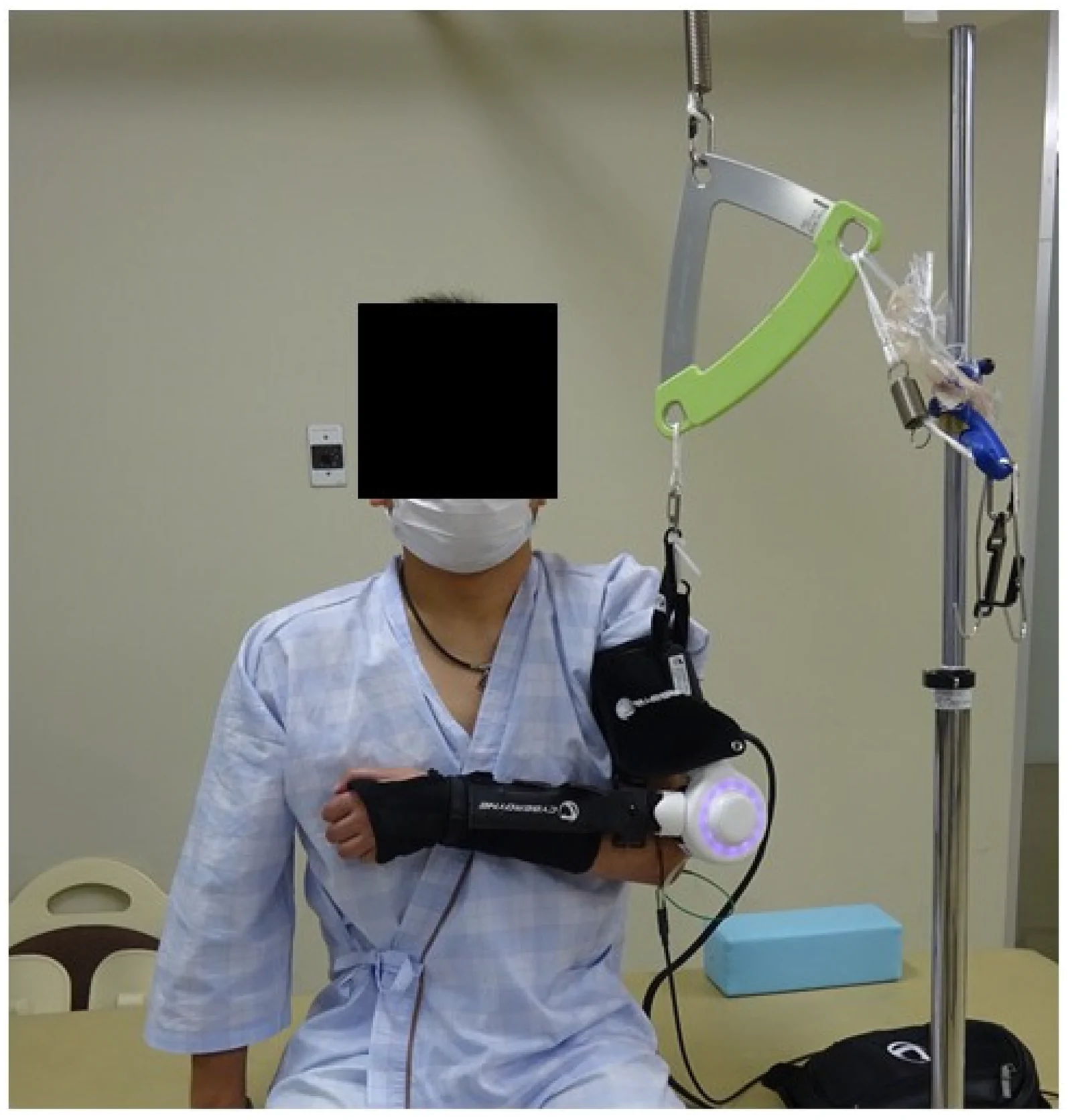
Elbow flexion training using an upper-limb hybrid assistive limb was performed.

The upper-limb HAL is composed of an actuator, a control device, a handy controller, an arm attachment, a forearm attachment, HAL surface electrode sensors, a suspension device, and a battery [24]. It is hung by a suspending device and worn on the elbow such that the actuator is positioned on the lateral elbow. The HAL surface electrodes are attached to the biceps and triceps brachii. The upper-limb HAL device cannot be worn alone; it requires one assistant and takes approximately 3 min. Upper-limb HAL-assisted elbow rehabilitation was introduced during the early post-reinnervation phase of BPI recovery (Figure 1). Training sessions focused on elbow flexion exercises using the upper-limb HAL and were scheduled from once weekly to once monthly, primarily in the outpatient setting. Each rehabilitation session lasted approximately 60 min and included vital sign monitoring, evaluations before and after training, and the HAL-assisted exercise program. During each session, patients were encouraged to perform as many elbow flexion repetitions as possible with HAL assistance, although the total number of repetitions was modified based on individual fatigue levels and motivation. In a seated position, patients performed approximately 20–100 elbow flexion exercises per session using the upper-limb HAL. During these exercises, an operator or therapist operated the controller and supported the device. For outpatients, the recommended training schedule ranged from one session weekly to one session every 2 weeks, although the final frequency was determined considering the availability and convenience of each patient.

All five patients who underwent ICN-MCN transfer began upper-limb HAL training when the biceps brachii strength reached MRC grade 1 after re-innervation following surgery. Elbow flexion strength was assessed before starting and after completing the HAL program, as well as at the final follow-up (mean: 28.0 months). The time required to achieve a biceps brachii strength of MRC grade 3 was also recorded.

### 2.5. Measurement of Surface EMG

All five patients who underwent ICN-MCN transfer and started upper-limb HAL training when their biceps brachii strength was MRC grade 1, all five were evaluated using a wireless surface EMG system (Clinical DTS, Noraxon, Inc., Scottsdale, AZ, USA, and SAKAI Medical Co., Ltd., Tokyo, Japan).

### 2.6. Outcome Measures

#### 2.6.1. Measurement Procedure of Surface EMG

**Figure 2:**
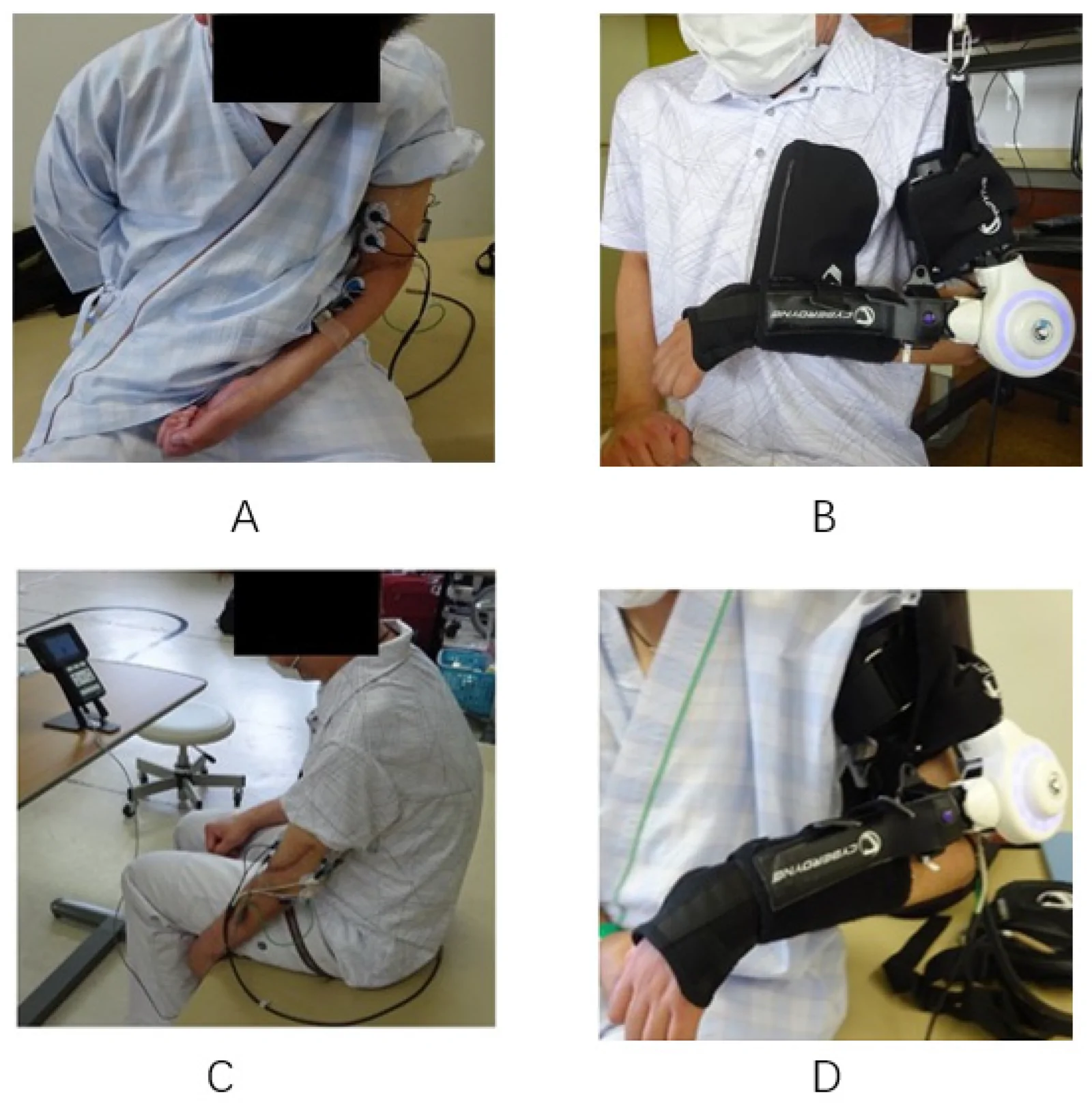
Electromyography (EMG) was performed under four conditions in five patients. The mean biceps amplitude was measured during elbow flexion (Medical Research Council [MRC] grades 1 and 2) as follows: (**A**) without hybrid assistive limb (HAL), (**B**) with HAL, (**C**) during conventional biofeedback (BF) training using a device without HAL, and (**D**) during 5 s of elbow flexion with HAL. The mean amplitude was compared between conditions (**A**,**B**) and between conditions (**C**,**D**). Specifically, (**A**) patients performed elbow flexion (MRC grade 1 or 2) without HAL. (**B**) Elbow flexion with the HAL. (**C**) Conventional BF training using a device without an HAL. (5 s of elbow flexion, MRC grade 1 or 2). (**D**) Five seconds of elbow flexion with HAL.

Five patients who underwent ICN-MCN transfer underwent surface EMG evaluation using a wireless EMG system. Electromyographic assessment was performed at the beginning of each session using a wireless EMG system to record biceps brachii muscle activity. Conventional BF training using a device (Myotrace 400, Noraxon, Inc., Scottsdale, AZ, USA and SAKAI Medical Co., Ltd., Tokyo, Japan) was performed in the work-rest mode for 5 s of elbow flexion (work) and 10 s of rest. This work-rest cycle was repeated 10 times at the end of each session. The mean biceps amplitude during elbow flexion was measured under four conditions: with and without HAL (Figure 2A,B), during conventional BF training using a device without HAL (Figure 2C), and 5 s of elbow flexion with HAL (Figure 2D). Specifically, the mean amplitudes of the biceps with and without HAL were compared, as were the amplitudes during conventional BF training without HAL and 5 s of elbow flexion with HAL.

#### 2.6.2. Wireless Surface EMG Device System

Five patients who underwent ICN-MCN transfer and began upper-limb HAL training at MRC grade 1 were also evaluated using surface EMG. Biceps activity (mean amplitude) was measured with surface EMG during upper-limb HAL training in patients with a biceps brachii strength of MRC grade 1 or 2. The measurement system comprised a one-channel wireless surface EMG system (Noraxon, Inc., Scottsdale, AZ, USA) and bipolar Ag-AgCl surface electrodes. The sampling rate was 2000 Hz, and the signal was filtered (Butterworth band-pass filter, 10–500 Hz). To reduce impedance, the skin was wiped with alcohol before applying the electrodes. Pairs of surface electrodes (1-cm diameter, 2.5-cm center-to-center spacing) were applied to the biceps muscle of the affected limb. Surface EMG recordings were obtained in accordance with the recommendations of the Surface ElectroMyoGraphy for the Non-Invasive Assessment of Muscles (SENIAM) project [26]. These European guidelines were established to standardize procedures related to electrode selection, placement, signal processing, and modeling for non-invasive muscle assessment. The EMG signals were processed and normalized based on the maximal voluntary contraction method. We instructed the patient to perform maximum elbow flexion (best effort) on the affected limb and considered this to be 100%.

### 2.7. Statistical Analysis

The Wilcoxon signed-rank test was used to evaluate the differences between the HAL and conventional BF during EMG evaluation in five patients. All statistical analyses were conducted using IBM SPSS Statistics version 22 (IBM, Armonk, NY, USA). Statistical significance was set at *p* < 0.05.

## 3. Results

### 3.1. ICN-MCN Transfer in Five Patients

Elbow training with upper-limb HAL began at an average of 8.6 postoperative months in the five patients who underwent ICN-MCN transfer. HAL training finished at 24.0 postoperative months, resulting in a mean duration of 15.4 months. Overall, a mean of 24.0 (range, 11–43) HAL sessions were performed. At the start of HAL, all five patients had a biceps brachii strength of MRC grade 1. At HAL finish, two (40.0%) patients had a strength of MRC grade 2 and three (60.0%) had a strength of MRC grade 3. At the final assessment (mean: 28.0 months), one (20.0%) patient had a strength of MRC grade 2, three (60.0%) had a strength of MRC grade 3, and one (20.0%) had a strength of MRC grade 4 (Table 1).

### 3.2. Surface EMG in Five Patients

**Figure 3:**
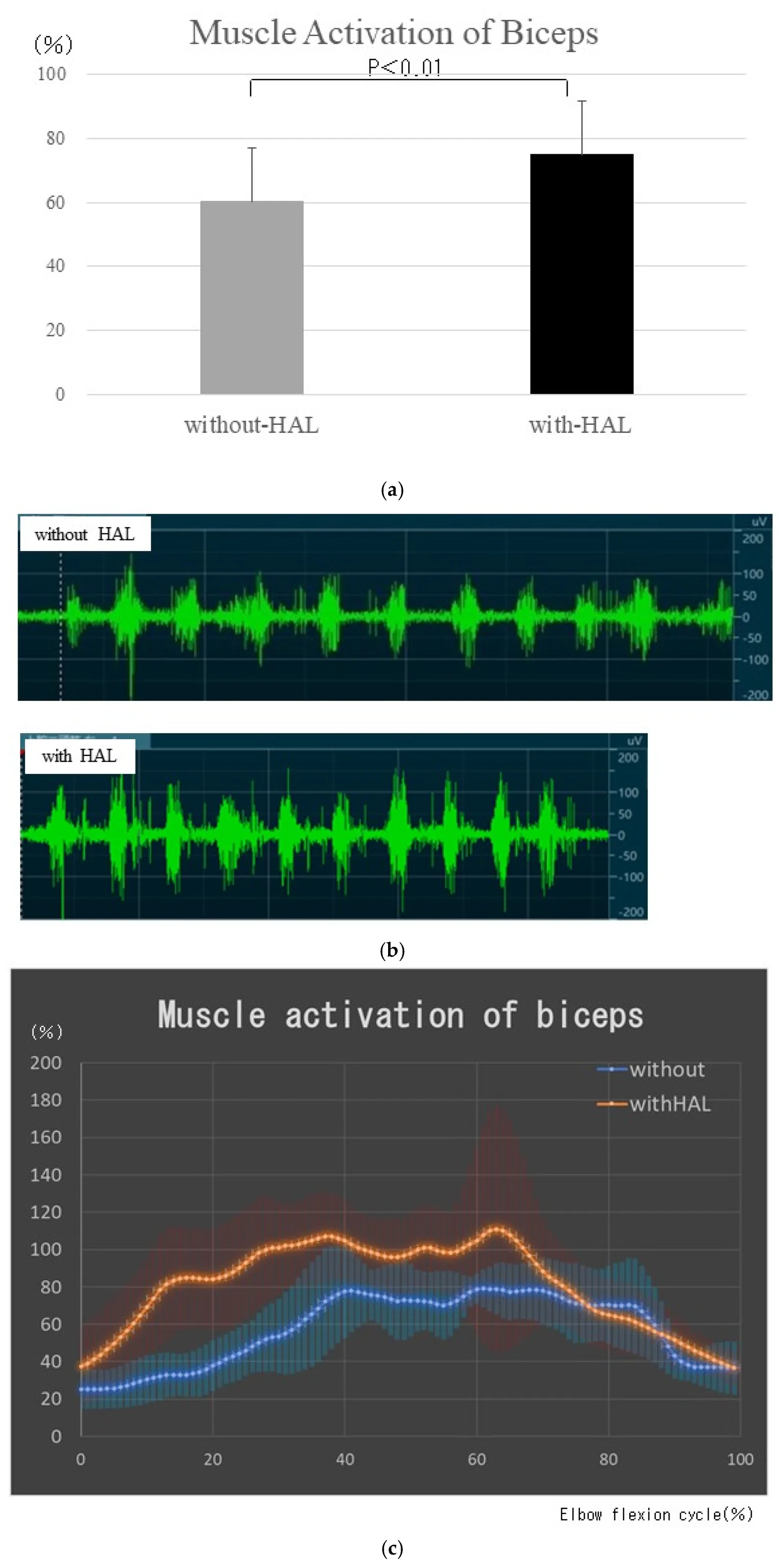
Electromyography (EMG) for biceps muscle activities with and without hybrid assistive limb (HAL). (**a**) The mean biceps amplitude during elbow flexion in five patients. The biceps muscle activity was significantly higher with HAL than without HAL. (**b**) Raw EMG waveform of the biceps, 10 repetitions of elbow flexion with and without HAL in case 1 (HAL session 5). (**c**) EMG traces of biceps muscle activity during 10 repetitions of elbow flexion with and without HAL in case 1 (HAL session 5). (**b**,**c**) show the EMG activity of the biceps muscle in the same patient (case 1).

Five patients in the ICN–MCN transfer group (Table 1) underwent muscle activity assessment using the EMG system. Compared with maximal voluntary contraction, the mean biceps amplitudes during elbow flexion in the five patients were 60.3 ± 16.7% without HAL and 74.9 ± 22.7% with HAL (Figure 3a). Representative EMG waveforms during elbow flexion in case 1 are shown in Figure 3b,c. Figure 3b,c show the EMG activity of the biceps muscle in the same patient (case 1).

**Figure 4:**
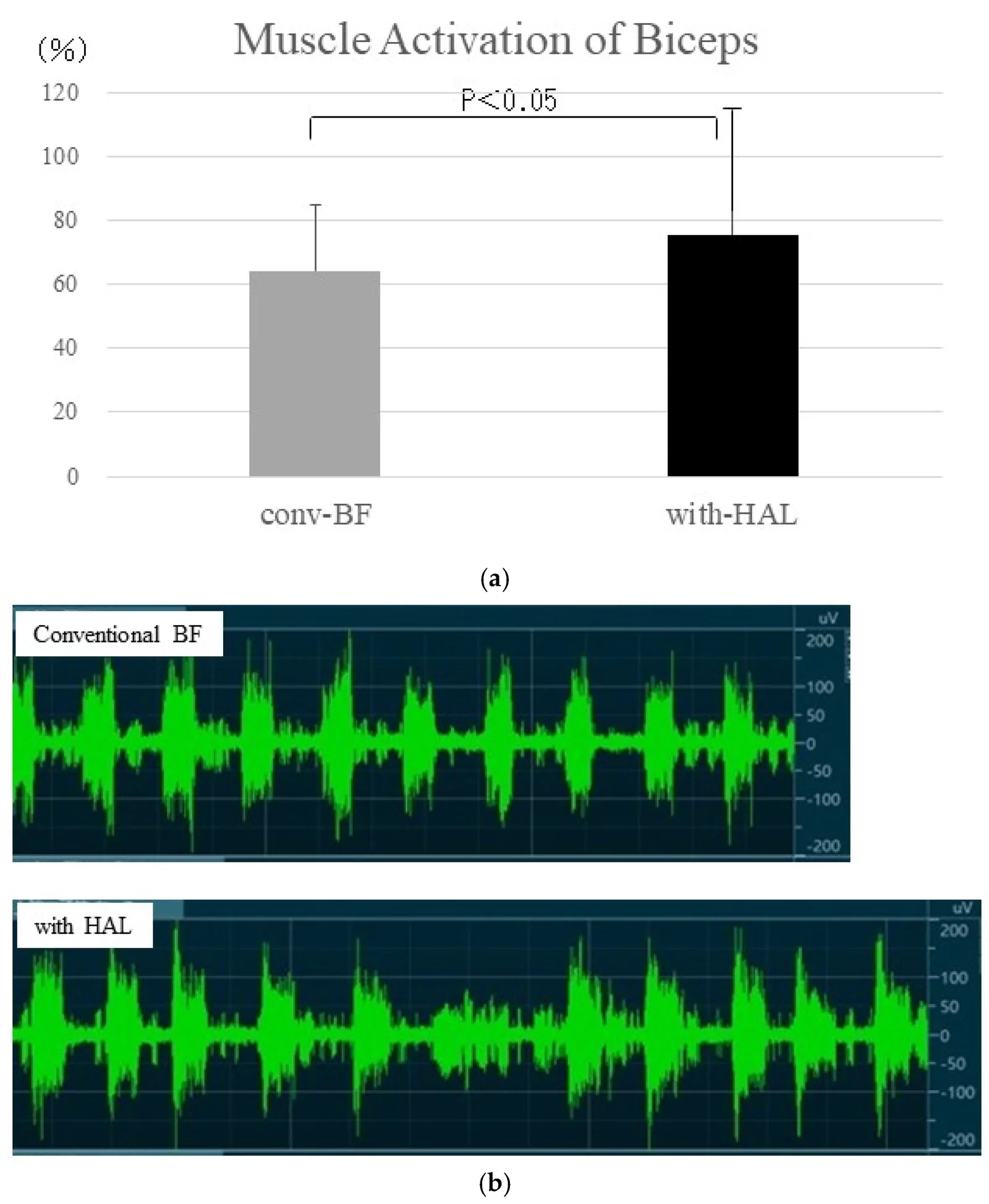

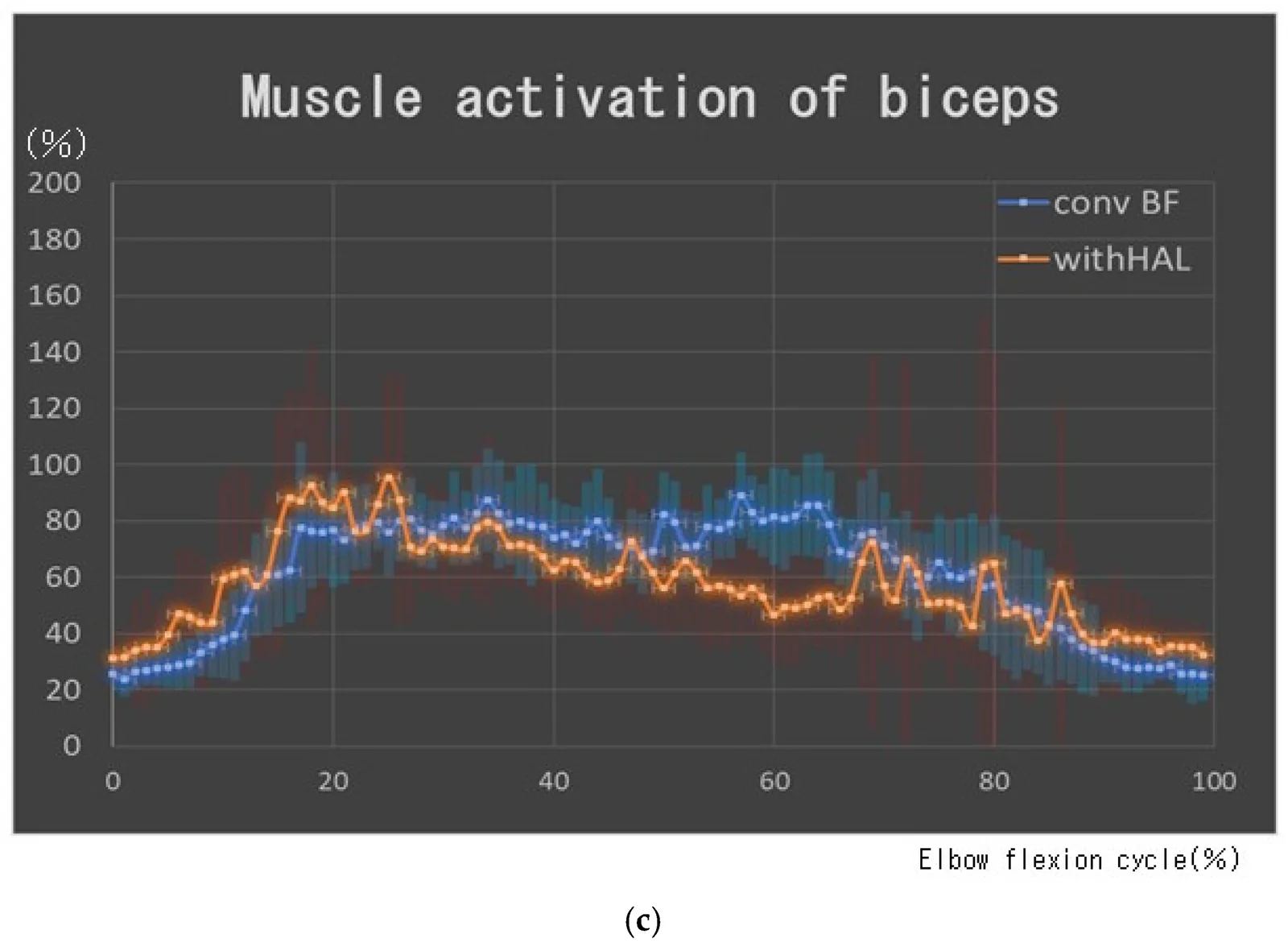
Electromyography (EMG) for biceps muscle activities during conventional biofeedback (BF) training using a device and with hybrid assistive limb (HAL). (**a**) The mean biceps amplitude during 5 s of elbow flexion in five patients. The biceps muscle activity was significantly higher with HAL than with conventional BF training using a device. (**b**) Raw EMG waveform of the biceps during 10 repetitions of 5-s elbow flexion with conventional BF training using a device and with HAL in case 1 (HAL session 5). (**c**) EMG traces of biceps muscle activity during 10 repetitions of elbow flexion with conventional BF training using a device and with HAL in case 1 (HAL session 5).

During conventional BF training using a device without HAL and during 5 s of elbow flexion with HAL, the mean biceps amplitudes were 64.3 ± 20.4% and 75.6 ± 39.3%, respectively (Figure 4a). The biceps muscle activity was significantly higher with HAL than during conventional BF training without HAL (*p* = 0.028). Representative EMG waveforms for case 1 are shown in Figure 4b,c. Overall, the mean biceps amplitudes measured under HAL-assisted conditions were significantly higher than those measured without HAL.

## 4. Discussion

Our study suggested that BF training with the newly developed upper-limb HAL has the potential to be a high-quality electromyographic training method following brachial plexus reconstruction surgery. The upper-limb HAL can detect biceps brachii bioelectric signals and induce early joint movement, allowing the separation of elbow flexion and extension. Repeated practice of this separation can prevent co-contraction. Furthermore, repeated elbow joint movements coordinated with muscle activation during the early postoperative period can promote neuroplastic changes in the corticospinal pathway, which activate spinal motor neurons innervating the intercostal nerve, thus enabling the acquisition of useful elbow flexion.

**Figure 5:**
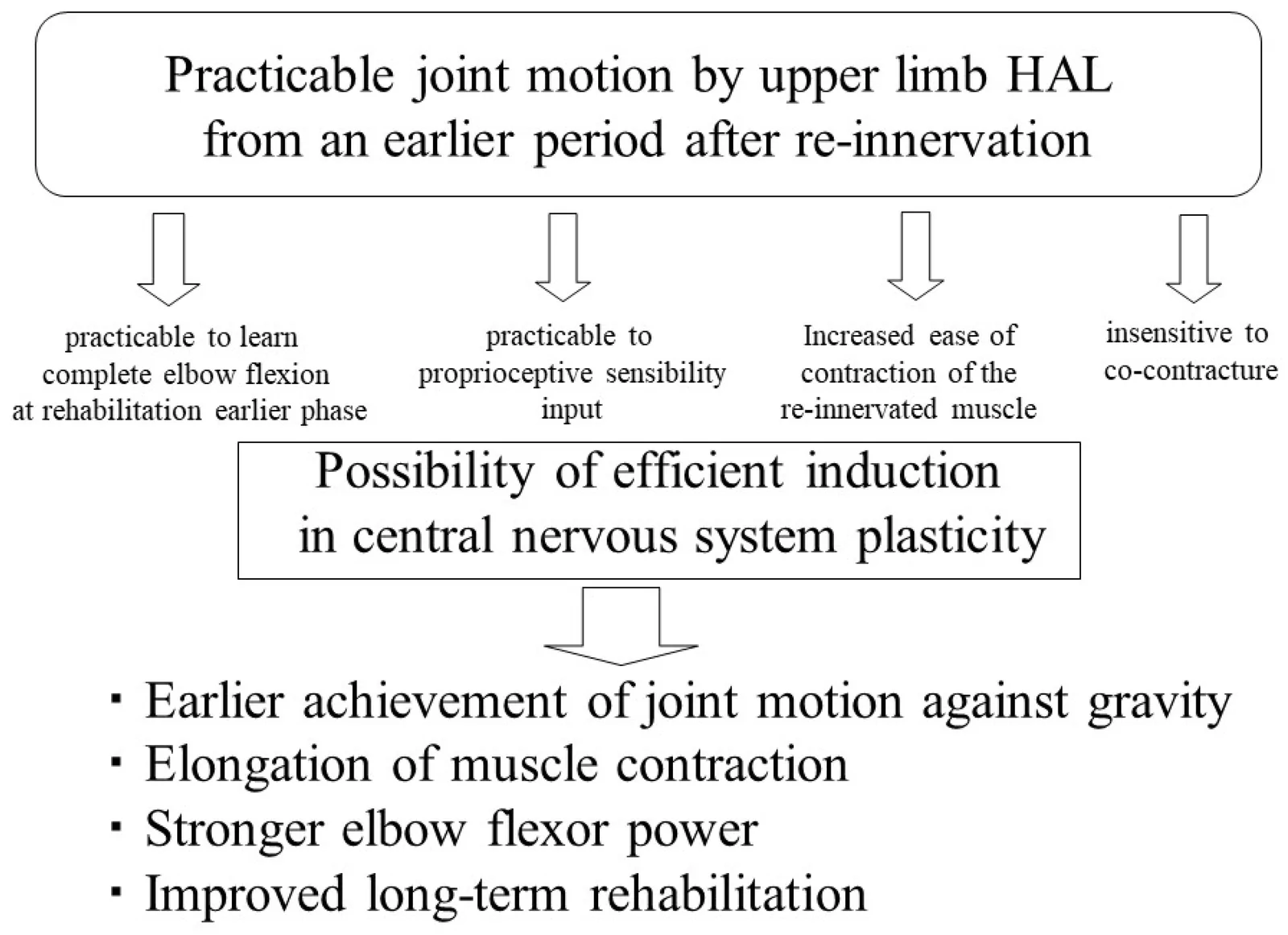
Hypothesis and possibility for robotic BF technology using upper-limb HAL following elbow flexor reconstruction after BPI.

After functional reconstruction in BPI, upper-limb HAL training can be initiated immediately after re-innervation is achieved (elbow flexor strength, MRC grade 1). This approach is expected to enable early learning of complete elbow flexion, enhance proprioceptive sensibility input from the elbow joint, and facilitate contraction of the re-innervated muscles through HAL-assisted flexion. Furthermore, this approach may help decrease the tendency toward co-contraction in patients with upper-type palsy. We speculate that these mechanisms, combined with enhanced central nervous system plasticity and more efficient facilitation of movement, played an important role in the positive results obtained in the present study. Although the following explanation and Figure 5 represent that our proposed hypothesis were not directly tested in the present study, upper-limb HAL training may therefore allow earlier achievement of antigravity joint motion, prolonged muscle contraction, stronger elbow flexor strength, and improved long-term rehabilitation outcomes.

Biceps brachii activity was significantly higher when patients wore the upper-limb HAL, enabling active elbow flexion with afferent contraction, than when patients attempted movements without the HAL, which resulted in isometric contraction without joint motion (Figure 3a). Likewise, biceps activation during 5 s of HAL-assisted elbow flexion was significantly higher than that during conventional BF training using a device alone (Figure 4a). These results suggest that upper-limb HAL training is the BF training with high electromyographic quality. Early facilitation of elbow flexion after re-innervation may synchronize central nervous system motor activity with limb movements, thereby enhancing neuroplasticity.

This study has some limitations, including its small sample size, non-randomized design, and electrophysiological evaluation in only a subset of patients; functional outcomes (e.g., Disability of the Arm, Shoulder, and Hand score, active range of motion, or patient-reported outcomes) beyond muscle strength were not systematically assessed. In addition, the effects of conventional rehabilitation were included in this study, and further studies are needed to determine the true effects of HAL training. Additionally, the results in this study are preliminary and need confirmation in larger, multicenter trials. Another limitation of this study is its limited generalizability, owing to the heterogeneity in training frequency and total number of training sessions among participants. Regarding Figure 3b, a discrepancy in the timing of muscle contractions was observed between the without-HAL and with-HAL conditions. Since this indicates that the timing of the motor task was not standardized between the two conditions, we consider this to be one of the limitations of the present study. Future studies with larger cohorts and randomized controlled trials comparing the HAL and conventional BF approaches are required.

## 5. Conclusions

Elbow flexion BF training using a robotic upper-limb HAL may be a high-quality electromyographic training method after elbow flexor reconstruction for BPI.

## Figures and Tables

**Table 1:** Clinical characteristics and Medical Research Council grade of the biceps for all patients with brachial plexus injury who received upper-limb hybrid assistive limb training.

Case No	Age	Sex	Right/Left	Diagnosis	Operation	Duration Since Surgery (HAL Start)	HAL Finish (POM)	Duration of HAL	Total HAL Sessions	MRC Grade of Biceps						Reference
										HAL Start (POM)	HAL Finish (POM)	Final Assessment (POM)	Achievement of MRC Grade [1]	Achievement of MRC Grade [2]	Achievement of MRC Grade [3]	
1	20	M	L	BPI, whole type	Elbow flexor reconstruction (ICN transfer), finger flexor reconstruction (LD transfer)	9 mo	25 mo	16 mo	43	1 (9)	3 (25)	3 (24)	8 mo	12 mo	24 mo	[24]
2	53	M	L	BPI, whole type	Elbow flexor reconstruction (ICN transfer)	9 mo	24 mo	15 mo	27	1 (9)	3 (24)	3 (32)	8 mo	13 mo	23 mo	[24]
3	19	M	R	BPI, C5–C8 preganglionic injury	Elbow flexor reconstruction (ICN transfer)	8 mo	28 mo	20 mo	11	1 (8)	2 (28)	2 (34)	8 mo	18 mo	NA	[24]
4	55	M	L	BPI, whole type	Elbow flexor reconstruction (ICN transfer)	8 mo	21 mo	13 mo	19	1 (8)	2 (21)	3 (26)	7 mo	9 mo	26 mo	[24]
5	39	M	L	BPI, whole type	Elbow flexor reconstruction (ICN transfer)	9 mo	22 mo	13 mo	20	1 (9)	3 (22)	4 (24)	7 mo	12 mo	17 mo	[24]
mean	37.2					8.6	24.0	15.4	24.0	(8.6)	(24.0)	(28.0)	7.6	12.8	22.5	

BPI: brachial plexus injury; HAL: hybrid assistive limb; ICN: intercostal nerve; LD: latissimus dorsi muscle; MRC: Medical Research Council; POM: postoperative months.

## Data Availability

The data supporting the findings of this study are available within the article. Additional information may be requested from the corresponding author.

## Abbreviations

The following abbreviations are used in this manuscript:

BF	Biofeedback
BPI	Brachial plexus injury
CIME	Center for Innovating Medicine and Engineering
DTS	Direct Transmission System
EMG	Electromyography
HAL	Hybrid assistive limb
MRC	Medical Research Council
WMA	World Medical Association
